# A mode of CVVH with regional citrate anticoagulation compared to no anticoagulation for acute kidney injury patients at high risk of bleeding

**DOI:** 10.1038/s41598-019-42916-1

**Published:** 2019-04-29

**Authors:** Jianping Gao, Feng Wang, Yonggang Wang, Dan Jin, Liping Tang, Konghan Pan

**Affiliations:** Critical Care Department, Sir Run Run Shaw Hospital, Zhejiang University School of Medicine, Hangzhou, 310016 China

**Keywords:** Acute kidney injury, Continuous renal replacement therapy, Outcomes research, Continuous renal replacement therapy

## Abstract

The study was designed to assess a practical mode of postdilution continuous venovenous hemofiltration (CVVH) with regional citrate anticoagulation (RCA) using a calcium-containing replacement solution, and to compare it with a CVVH mode with no anticoagulation (NA). Both methods were employed in our center for acute kidney injury (AKI) patients at high risk of bleeding. Fifty-six patients were equally allocated into the RCA-CVVH group and the NA-CVVH group. The study displayed no significant differences between groups involving baseline characteristics, severity level, blood gas analysis, hepatic/renal/coagulative functions, electrolytes, hemoglobin concentration, and platelet counts before or after continuous renal replacement therapy (CRRT). Compared to the NA-CVVH group, the RCA-CVVH group had a lower level of transfused packed red blood cells and platelet as well as a longer filter lifespan. The result showed no substantial differences between groups in terms of the mean supporting time and cost involving CRRT per person, the length of ICU and hospital stays, and the ICU survival. Homeostasis was basically preserved at a target range during the RCA post-CVVH procedure. Serious complications did not arise during the RCA process. RCA postdilutional CVVH is a safe and effective mode for application in AKI patients with a high risk of bleeding, and it can extend the filter lifespan and decrease blood loss, compared with the NA mode for CRRT. Further studies are needed to evaluate this mode for CRRT. (Retrospective Registration number ChiCTR1800016462, Registration date 2/6/2018)

## Introduction

According to the Kidney Disease Improving Global Outcomes (KDIGO) definition of acute kidney injury (AKI), the general incidence of adult AKI is 19–24%, and the mortality attributable to it is 22–26%^1^. Based on the epidemiology of AKI in China, the incidence of AKI ranges between 5% and 50%. The incidence of intensive care unit (ICU) stay is 20% to 50%, and the AKI mortality is up to 50%^2^. Moreover, AKI is commonly associated with a longer hospital length of stay and higher medical costs^3^.

Renal replacement therapy (RRT) is usually applied in critical AKI patients in the ICU. Continuous RRT (CRRT) has an advantage over intermittent RRT when the AKI patient is hemodynamically unstable, has cerebral edema or acute brain injury^4,5^. Appropriate anticoagulation is an important part of the CRRT procedure. Many factors associated with high risks of bleeding exist in the ICU, such as post-major surgeries involving the heart, brain or abdomen, coagulopathy and/or thrombocytopenia induced by trauma, sepsis or active bleeding, and combined therapy with anticoagulant, antiplatelet or thrombolytic drugs^6,7^.

The KDIGO guideline recommends that the regional citrate anticoagulation (RCA) mode should be the first choice for CRRT in a patient without contraindications for citrate. Generally, the guideline also recommends RCA rather than no anticoagulation (NA) for CRRT in AKI patients with a high risk of bleeding. However, a comparative study between these two modes is still limited^8,9^. Based on local ICU resources, the NA or RCA mode could be chosen for CRRT if the patient has a high risk of bleeding, which was suggested by the domestic guideline of CRRT application from the Chinese Society of Critical Care Medicine. Due to a shortage of dedicated RCA-CRRT machines and commercial 4% or 5% sodium citrate, and especially an absence of commercial calcium-free dialysate, the RCA mode has not been employed extensively for CRRT in China. The NA mode is commonly the first choice for CRRT in most domestic centers when a high risk of bleeding exists. The RCA postdilutional CVVH (post-CVVH) mode using the Fresenius CiCa^®^ system with a commercial calcium-containing replacement fluid has been adopted for use in our center.

Therefore, an open randomized controlled trial (RCT) was designed to assess this practical mode of RCA post-CVVH using a calcium-containing replacement fluid and to compare it to the NA mode, both of which were employed in our center in AKI patients with a high risk of bleeding.

## Results

The comparison of the characteristics of patient with AKI who had a high risk of bleeding is displayed in Table 1. These patients underwent CRRT with the RCA or NA mode. No significant differences in statistics were concluded between the two groups in terms of age, sex, comorbidities including hypertension, type 2 diabetes mellitus and chronic renal dysfunction, and patient source (postsurgery or medical ward). Most of the patients were elderly and were admitted to the ICU after major surgery. The severity of critical illness was similar between the RCA and NA groups using the ICU scoring systems, such as APACHE II and SOFA. There were no significant differences between the two groups before or after CRRT in blood pH, the blood levels of bicarbonate ion (HCO_3_^−^), base excess, lactate, total bilirubin, alanine aminotransferease, aspartate aminotransferase, creatinine, blood urea nitrogen, prothrombin time (PT), activated partial thromboplastin time (APTT), hemoglobin, platelet count, sodium (Na), magnesium (Mg) and total calcium (tCa).

**Table 1:** Characteristics of AKI patients with a high risk of bleeding for CRRT under the two different anticoagulation modes.

Characteristics	RCA group	NA group	*P*
age (year)	60.4 (53.8–67.0)	65.2 (60.7–69.8)	0.223
male (%)	67.9 (19/28)	60.7 (17/28)	0.577
weight (kg)	70.2 (66.9–73.8)	71.5 (68.5–74.5)	0.582
hypertension (%)	50.0 (14/28)	57.1 (16/28)	0.592
type 2 diabetes mellitus (%)	21.4 (6/28)	17.9 (5/28)	0.737
chronic renal dysfunction (%)	25.0 (7/28)	17.9 (5/28)	0.515
postsurgery (%)	71.4 (20/28)	82.1 (23/28)	0.342
APACHE II	20.6 (18.3–23.0)	19.7 (17.7–21.8)	0.541
SOFA	12.3 (11.2–13.4)	11.9 (10.6–13.3)	0.672
pro-pH	7.24 (7.20–7.27)	7.22 (7.19–7.26)	0.512
pro-HCO_3_^−^ (mmol/L)	19.1 (17.8–20.4)	18.5 (17.1–19.8)	0.513
pro-BE (mmol/L)	−6.0 ([−6.5]–[−5.4])	−5.7 ([−6.1]–[−5.3])	0.415
pro-Lac (mmol/L)	6.8 (5.8–7.9)	7.6 (6.5–8.6)	0.298
pro-tBil (μmol/L)	33.1 (25.4–40.8)	39.1 (34.1–44.0)	0.188
pro-ALT (U/L)	77.5 (59.0–89.0)	101.5 (74.5–112.5)	0.129
pro-AST (U/L)	85.0 (64.0–102.0)	66.0 (58.0–86.5)	0.187
pro-Cr (μmol/L)	200.5 (172.8–228.3)	227.1 (201.3–252.9)	0.156
pro-BUN (mmol/L)	16.0 (13.1–18.9)	14.2 (11.4–16.9)	0.347
pro-PT (s)	20.6 (18.8–22.4)	22.0 (20.6–23.4)	0.219
pro-APTT (s)	50.9 (47.3–54.6)	54.3 (50.2–58.3)	0.222
pro-Hb (g/dl)	8.5 (8.0–9.1)	8.2 (7.7–8.6)	0.287
pro-Plt (10^9^/L)	64.1 (50.7–77.5)	81.5 (60.8–102.2)	0.153
pro-Na (mmol/L)	144.1 (141.8–146.5	146.2 (144.3–148.2)	0.168
pro-Mg (mmol/L)	0.96 (0.93–1.00)	0.92 (0.88–0.97)	0.170
pro-tCa (mmol/L)	2.15 (2.09–2.20)	2.12 (2.07–2.17)	0.521
post-pH	7.39 (7.37–7.41)	7.38 (7.35–7.40)	0.478
post-HCO_3_^−^ (mmol/L)	23.3 (22.7–23.9)	23.5 (23.0–24.0)	0.576
post-BE (mmol/L)	−0.7 ([−1.5]−0.1)	−1.1 ([−2.1]–[0.2])	0.470
post-Lac (mmol/L)	2.6 (2.0–3.2)	3.4 (2.8–3.9)	0.068
post-tBil (μmol/L)	29.3 (22.9–30.7)	27.1 (23.5–36.4)	0.718
post-ALT (U/L)	68.0 (58.0–75.0)	56.0 (48.5–60.0)	0.082
post-AST (U/L)	61.5 (52.5–71.0)	71.0 (54.0–76.5)	0.431
post-Cr (μmol/L)	97.2 (86.6–107.8)	101.7 (89.5–113.9)	0.567
post-BUN (mmol/L)	5.98 (5.29–6.67)	5.58 (4.92–6.25)	0.402
post-PT (s)	14.0 (12.4–15.6)	15.6 (14.7–16.5)	0.080
post-APTT (s)	40.0 (36.8–43.3)	41.3 (38.1–44.5)	0.574
post-Hb (g/dl)	8.6 (8.2–9.0)	8.1 (7.7–8.5)	0.082
post-Plt (10^9^/L)	57.0 (50.0–61.0)	50.0 (44.0–55.0)	0.268
post-Na (mmol/L)	140.8 (138.3–143.2)	143.5 (140.9–146.1)	0.122
post-Mg (mmol/L)	0.90 (0.84–0.95)	0.94 (0.89–0.99)	0.213
post-tCa (mmol/L)	2.23 (2.17–2.30)	2.19 (2.11–2.27)	0.396
effluent rate (ml/kg/h)	22.8 (21.6–23.8)	23.4 (22.4–24.5)	0.379
CRRT supporting time (h)	92.9 (76.2–109.7)	97.1 (82.7–111.6)	0.700
CRRT expense ($)	2474.6 (2053.3–2895.9)	2585.7 (2248.6–2922.9)	0.674
length of ICU stay (d)	8.2 (6.6–9.7)	9.1 (7.3–10.9)	0.425
length of hospital stay (d)	14.9 (11.9–17.9)	17.1 (13.7–20.6)	0.318
ICU survival (%)	57.1 (16/28)	46.4 (13/28)	0.422

Abbreviation: AKI: Acute Kidney Injury, ALT: Alanine Aminotransferease, APACHE II: Acute Physiology and Chronic Health Evaluation, APTT: Activated Partial Thromboplastin Time, AST: Aspartate Aminotransferase, BE: Base Excess, BUN: Blood Urea Nitrogen, Cr: Creatinine, CRRT: Continuous Renal Replacement Therapy, Hb: Hemoglobin, ICU: Intensive Care Unit, Lac: Lactate, NA: No Anticoagulation, Plt: Platelet, PT: Prothrombin Time, RCA: Regional Citrate Anticoagulation, SOFA: Sequential Organ Failure Assessment, tBil: Total Bilirubin, tCa: Total Calcium.

**Figure 1:**
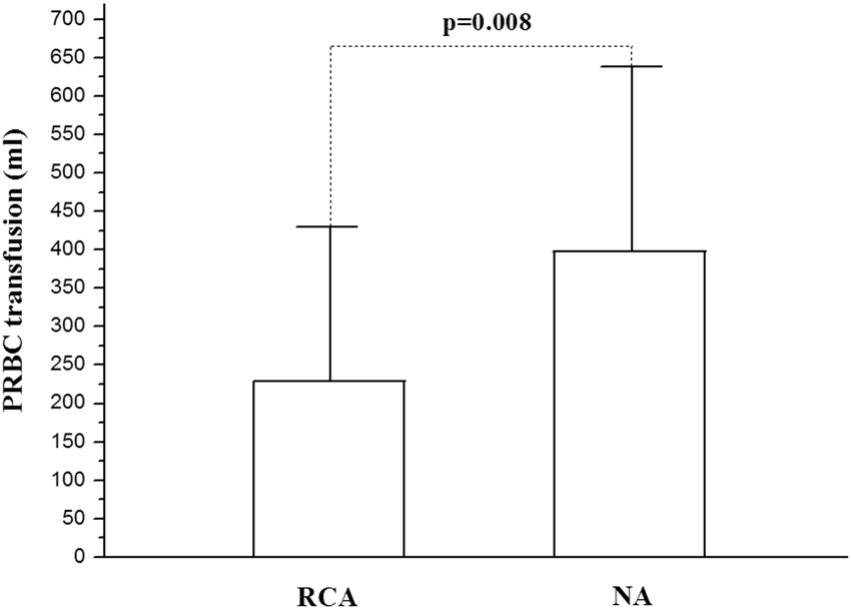
The volume of packed red blood cells (PRBC) transfused during continuous renal replacement therapy (CRRT). Less PRBC volume was needed in the citrate anticoagulation (RCA) mode compared with that in the no anticoagulation (NA) mode.

**Figure 2:**
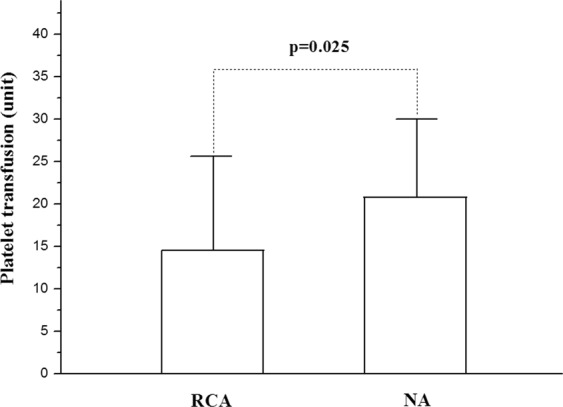
The volume of platelet transfusion during continuous renal replacement therapy (CRRT). Less platelet volume was needed in the citrate anticoagulation (RCA) mode compared with that in the no anticoagulation (NA) mode.

**Figure 3:**
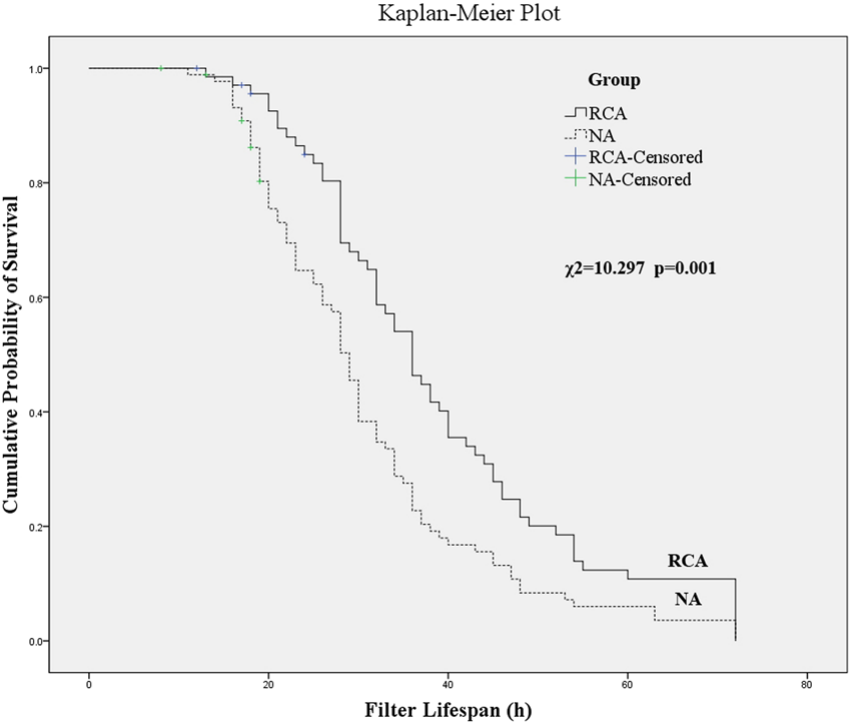
The Kaplan-Meier survival analysis using the log-rank test (Mantel-Cox test) for comparing the hemofilter lifespan between regional citrate anticoagulation (RCA) and no anticoagulation (NA) modes. The filter lifespan was relatively longer in the RCA mode compared with that in the NA mode.

The volume of PRBC transfusion needed during the RCA-CRRT was 228.9 ± 197.7 ml, which was comparatively less than that needed during the NA-CRRT (393.7 ± 244.5 ml) (Fig. 1). The volume of platelet infusion during the RCA-CRRT was 14.4 ± 10.8 unit, which was also less than that needed during the NA-CRRT (20.6 ± 9.4 unit) (Fig. 2). The median time of filter survival was 36.0(32.1–39.9) h for the RCA group compared with only 29.0(27.3–30.7) h for the NA group. The Kaplan-Meier survival analysis revealed that the filter lifespan was relatively longer using the RCA mode compared with that in the NA mode (Fig. 3).

**Figure 4:**
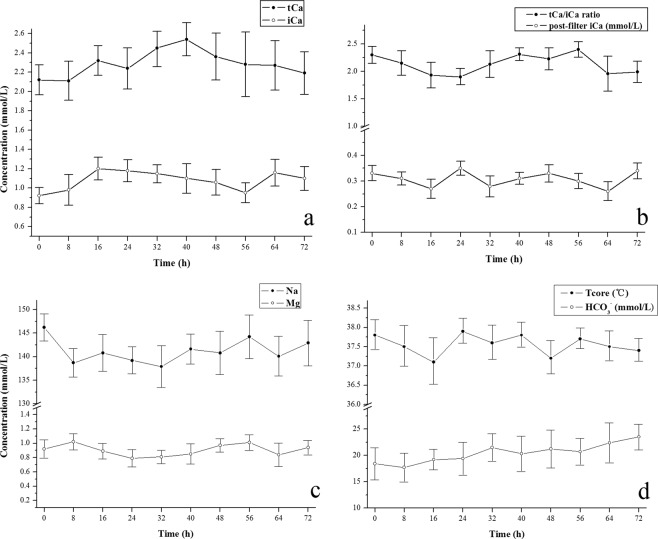
The trends of certain important parameters during continuous renal replacement therapy with regional citrate anticoagulation mode (RCA-CRRT). They are systemic blood concentrations of total calcium (tCa) and ionized calcium (iCa) (**a**), the ratio of tCa/iCa and the level of post-filter iCa in blood (**b**), the systemic blood concentrations of sodium (Na) and magnesium (Mg) (**c**), and the systemic blood level of bicarbonate ion (HCO3^−^) and the core temperature (Tcore) of the patients (**d**). The parameters are presented as the means ± standard deviation. Because of the varied filter survival results, a different number was finally reached at the fixed time-point of analysis with an 8-h interval during the CRRT. Relatively steady trends were concluded.

The trends of certain parameters during the RCA-CRRT procedure are shown in Fig. 4, including systemic levels of tCa, ionized calcium (iCa), Na, Mg, HCO_3_^−^, post-filter iCa, the ratio of tCa/iCa and the core temperature. The results generally fell within a normal target range, i.e., Na 137–147 mmol/L, Mg 0.75–1.02 mmol/L, tCa 2.11–2.52 mmol/L, iCa 1.12–1.32 mmol/L, post-filter iCa 0.25–0.35 mmol/L, HCO_3_^−^ 21.3–24.8 mmol/L, the ratio of tCa/iCa < 2.5 and the core temperature 36.5–37.8 °C. The trends were relatively steady.

**Figure 5:**
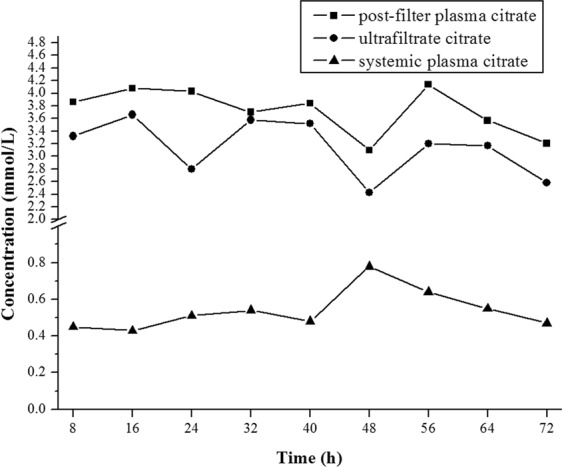
The citrate level in systemic and post-filter plasma and ultrafiltrate during continuous renal replacement therapy with the regional citrate anticoagulation mode. The citrate concentrations were relatively stable during the therapy.

The dynamic changes in citrate concentration in systemic and post-filter plasma and ultrafiltrate fluid during RCA-CRRT are shown in Fig. 5. Citrate levels were relatively stable during the extracorporeal therapy, with 0.45–0.62 mmol/L in systemic plasma, 2.69–3.48 mmol/L in the ultrafiltrate and 3.44–4.01 mmol/L in post-filter plasma. Serious complications did not arise during the RCA-CRRT process, such as critical hypo/hypernatremia, hypo/hypercalcemia, metabolic acidosis/alkalosis, major hemorrhage and malignant arrhythmia. The filtration fraction (FF) of the RCA-CRRT mode was about 33%.

The mean supporting time of CRRT per person was not significantly different between the groups of critical patients. The cost involving CRRT per person was a little less in the RCA group than that in the NA group ($2474.6 *vs*. $2585.7); however, a significant difference was not concluded. The lengths of the ICU and hospital stays both were shorter in the RCA group compared with those in the NA group; however, statistical difference was not achieved. The ICU survival was not ultimately improved in the RCA mode compared with that in the NA mode (Table 1).

## Discussion

A practical mode of RCA post-CVVH using calcium-containing replacement fluid was intentionally evaluated for its safety and efficiency and compared with that in the NA CVVH mode, both of which were adopted in our ICU AKI patients who had a high risk of bleeding. A lower volume of transfused PRBCs and platelets was needed during RCA-CRRT compared to that for NA-CRRT. A longer filter lifespan was also acquired using the RCA mode. Systemic concentrations of tCa, iCa, Na, Mg and HCO_3_^−^, post-filter iCa and the body core temperature were relatively steady during the RCA-CVVH procedure. The ratio of tCa/iCa was generally less than 2.5. Compared to the NA-CVVH mode, however, the RCA-CVVH mode rendered no benefits in cost and mean supporting time of CRRT, the length of the ICU and hospital stays, or the ICU survival of critical patients.

Due to the high incidence of AKI in critically ill patients, the CRRT machine has a standard configuration in secondary and tertiary hospitals in China. Concomitantly, its technique is being applied universally to the treatment of these patients in the ICU. CRRT could regulate the volume load, keep a balance of blood acid-base and electrolytes, and clear middle- and micromolecules, especially in hemodynamically unstable patients. If the filter of the extracorporeal system clots frequently, not only the efficiency and adequacy of CRRT will be affected but also blood coagulation factors and platelets will partially be consumed, which in turn could aggravate the bleeding and anemia risks. Moreover, the total amount of filter usage and its corresponding cost will be potentially increased.

Anticoagulation that is achieved via the systemic infusion of heparin is most widely employed for CRRT to avoid filter clotting and therapy interruption. However, systemic heparin anticoagulation has an associated risk of bleeding and heparin-induced thrombocytopenia. Recently, published studies have shown that RCA has some advantages over systemic heparin anticoagulation in prolonging filter runtime, reducing complications of organ bleeding, and potentially promoting patients’ survival^9,10^. RCA is also superior to the mode of regional heparin and protamine anticoagulation in reducing filter clotting and adverse events^11^. The regional heparinization mode is not recommended by the KDIGO guideline, even for patients with a risk of bleeding. The RCA mode for CRRT in China has gradually developed within recent years. Consequently, a practical and convenient protocol of RCA-CRRT should be explored, and the medical care personnel in the ICU must be educated and trained for the application of this technique.

Diverse RCA protocols were used in previous studies involving CRRT. There are different modalities including CVVHD, CVVH and CVVHDF^12–14^, different replacement solutions such as calcium-containing and calcium-free solutions^15,16^, citrate-containing and citrate-free solutions^17,18^, different infusion sites for replacement solutions such as pre- and post-filter sites^19,20^, and different methods of additional calcium supplementation, i.e., with and without calcium replacement^21,22^. In fact, the RCA mode without calcium replenishment renders a risk of hypocalcemia even with a calcium-containing replacement solution, including pre- and postdilutional CVVH modes^20,22^. Moreover, a predilutional CVVH mode with a calcium-containing replacement solution potentially increases the risk of citrate accumulation and even intoxication^22^. The citrate burden will also be augmented by the mode of RCA-CVVHDF with citrate- and calcium-containing dialysate and replacement solutions^17^.

The RCA post-CVVH mode with calcium-containing replacement solution and calcium replenishment was chosen in our present trial, partially because the commercial calcium-free dialysate was domestically unavailable. The Fresenius CiCa^®^ system has software for CVVHD and CVVHDF; however, these two modes generally need calcium-free dialysate. Moreover, manual production of calcium-free dialysate in the ICU circumstance could increase the risk of nosocomial infection of the solution and the workload of the nurses. Therefore, the RCA post-CVVH mode is intentionally explored using the CVVHD software within the Fresenius CiCa^®^ system. Compared with the predilution mode, postdilution could enhance CRRT efficacy of solute removal and lower the risk of citrate accumulation with RCA-CVVH using the calcium-containing replacement solution, although this mode might shorten the filter lifespan^23,24^. The FF of our RCA protocol (~33%) is exactly a little higher than that in other modes of CVVHD, CVVHDF, and predilutional CVVH with or without postdilutional solution. The FF in these protocols ranges approximately between 20% and 25%. However, the median time of the filter lifespan reaches 36.0 h in the present RCA post-CVVH protocol. Homeostasis within critical patients has also been maintained well under the protocol. Maybe, a bit high FF is allowed under RCA mode with the post-filter ionized calcium in therapeutic range (0.25–0.35 mmol/L). Anyhow, more studies are needed further to compare different RCA modes of CRRT.

Calcium citrate complexes can be formed when the citrate chelates iCa, part of which will be removed by the filter, while the rest of the chelate flows into the systemic circulation. The circulating chelate of citrate-calcium is metabolized into bicarbonate mainly in the liver and musculature, while the calcium ion will be rereleased into the circulation. Citrate accumulation may emerge when the citrate cannot be sufficiently metabolized in such conditions as severe liver failure or hypoperfusion within skeletal muscle. This phenomenon can be estimated indirectly through the blood tCa/iCa ratio because the blood citrate level is not routinely monitored clinically. The controversy still remains as to whether the RCA CRRT could be safely used in liver failure or hyperlactacidemia patients^12,25,26^. In any case, the blood electrolytes, including tCa and iCa, and blood gas analysis must be closely monitored if the RCA CRRT is employed in these critical patients.

The citrate accumulation did not arise during the RCA-CRRT process in our pilot study, with the ratio of tCa/iCa <2.5 and without refractory metabolic acidosis or alkalosis. The citrate level of systemic plasma for RCA post-CVVH during CRRT in our study was 0.45–0.62 mmol/L. In fact, the patients with severe liver failure in the trial were excluded. Further studies are needed involving absolute contradictions to RCA-CRRT, e.g., the extent of liver failure and hyperlactatemia. Perhaps lactate kinetics rather than initial severe hyperlactatemia is a better approach to assess the risk of citrate accumulation^27^.

Fibrin deposition occurs gradually on the surface of a hollow fiber membrane during the CRRT process, which will affect the filter patency and efficiency of the solute transmembrane clearance^24,28^. Therefore, some researchers recommend that the filter should be changed every 24 h to avoid a decrease in CRRT efficiency and the subsequent filter clotting. From our point of view, however, the filter lifespan should not be extended blindly beyond that which is recommended by the product instruction. The duration for continuous efficiency of filter function without clotting is 72 h, based on the patent product used in our study. The median time of the filter lifespan was 36.0 h in the RCA group, while the median time in the NA group was 29.0 h; the filter lifespan for both groups fell far below the usage limit of 72 h. Judged on the levels of blood electrolytes and renal function during CRRT, the filter performance was not considerably affected, although the filter lifespan was extended beyond 24 h in both groups. The cost of RCA-CRRT did not increase compared with that using the NA mode, although additional citrate fluid was consumed. Moreover, the expense for the total use of CRRT unit (filter & tubes) was reduced in the RCA mode as the filter lifespan was prolonged.

Our trial has some limitations. First, the sample in the study is relatively small, and therefore, a larger sample capacity is needed in future research. Second, other RCA modes (e.g., CVVHD and pre-CVVHDF) could also be undertaken and evaluated, especially if commercial calcium-free dialysate and replacement solution occur domestically in the future. After all, post-CVVH has a relatively high FF for CRRT (~33% for the present RCA mode). Third, the software for CVVHD was adopted for the CVVH procedure in the present trial because the Fresenius CiCa^®^ system only has software for CVVHD and CVVHDF. Suitable CVVH software is expected in the future for more convenient manipulation. Fourth, the CRRT machine only with Fresenius multiFiltrate^®^ has been adopted in our center. Other CRRT machines such as the Prismaflex^®^ Baxter can also be employed friendly for RCA post-CVVH. Studies comparing the effectiveness among the different CRRT devices are needed further.

In conclusion, RCA post-CVVH is a safe and effective mode for application in AKI patients with a high risk of bleeding. This mode can extend the filter lifespan and decrease blood loss compared with the NA mode for CRRT. However, the trial failed to show the benefits related to ICU survival and the length of ICU and hospital stays within the RCA mode. Further studies are needed to evaluate this mode of CRRT.

## Methods

This prospective pilot study was performed from January 2017 to January 2018 at the 90-bed adult mixed-ICU of the tertiary teaching hospital (Sir Run Run Shaw Hospital, SRRSH), Zhejiang University, China. The study adhered to the ethical principles for medical research involving human subjects according to the World Medical Association Declaration of Helsinki^29^ and was approved by the SRRSH Ethics Committee (No. 20170110-8). Informed consent was required from the patients, family/relative, guardian, or proxy according to Chinese law.

### Inclusion and exclusion criteria

The inclusion criteria were as follows: Stage 3 adult AKI patients according to the KDIGO guideline who had high risk factors for bleeding including post-major surgeries involving the heart, chest, abdomen, spine or brain, coagulopathy (PT or APTT > 1.5 times the normal control, or PT > 18 s, APTT > 60 s) and/or thrombocytopenia (<50 × 10^9^/L) induced by trauma, sepsis or active bleeding, and combined therapy with anticoagulant, antiplatelet or thrombolytic agents. The CRRT was initiated within 3 h after randomization. The exclusion criterion was severe liver failure (serum total bilirubin >171 μmol/L). If the ratio of tCa to iCa (tCa/iCa) in the blood exceeded 2.5, the RCA mode in CRRT was switched to the NA mode for fear of citrate accumulation.

### Instrumentation and solution

The following instruments and materials were applied in this study, including a blood gas analyzer (GEM^®^ premier 3000, Instrumentation Laboratory Company, Bedford, Massachusetts, USA) used in point-of-care testing, an automatic hematology analyzer (Beckman LH780, Atlanta, Georgia, USA), an automatic coagulation analyzer (Sysmex Ca-7000, Kobe, Hyogo, Japan), an automatic biochemistry analyzer (Dimension RxL Max-HM, Simens Corp., Munich, Germany), a vacuum blood collection tube (BD Vacutainer^®^ containing lithium heparin anticoagulant, Franklin Lakes, New Jersey, USA), two types of centrifuges (Sorvall Legend Mach 1.6R Centrifuge and Heraeus Fresco 21 microCentrifuge, Thermo Scientific™, Waltham, Massachusetts, USA), high-performance liquid chromatographic (HPLC) system (Waters Alliance 2695 Separations Module, Milford, Massachusetts, USA) and a CRRT machine for easy RCA implementation (multiFiltrate^®^, Fresenius Medical Care, Bad Homburg v.d.H., Germany).

The hemofilter (Ultraflux^®^ AV1000S) was composed of capillary fibers (Fresenius^®^ polysulfone) and markrolon cages, with an effective membrane surface area of 1.8 m^2^, a maximum transmembrane pressure (TMP) of 600 mmHg, lumen diameter of 220 μm and fiber wall thickness of 35 μm. All filters were patent at 72 h. Therefore, the filters and the corresponding connecting tubes were not changed routinely within this time period, unless clotting or a TMP > 300 mmHg developed in the extracorporeal circuit. The CRRT was discontinued if the patient died or withdrew from life-sustaining treatments. The lifespan of these filters was viewed as censored data in the following filter survival analysis.

**Figure 6:**
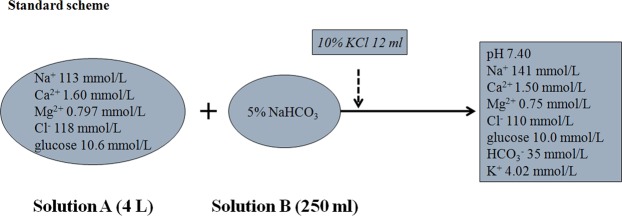
The components and their levels of the commercial calcium-containing replacement fluid. The standard scheme in our study was just for CRRT with no anticoagulation mode. A different infusion rate of Solution B (5% sodium bicarbonate) was used for CRRT with regional citrate anticoagulation.

Two commercial solutions were adopted in CRRT, i.e., a 4% trisodium citrate solution (200 ml, Nigale Biotechnology Co. Ltd., Sichuan, China) and the replacement fluid (Qingshan Likang, Pharmaceutical Co. Ltd., Chengdu, China). The replacement fluid contained solution A and solution B (5% sodium bicarbonate, NaHCO_3_), the standard scheme for which is shown in Fig. 6.

### CRRT protocol

**Figure 7:**
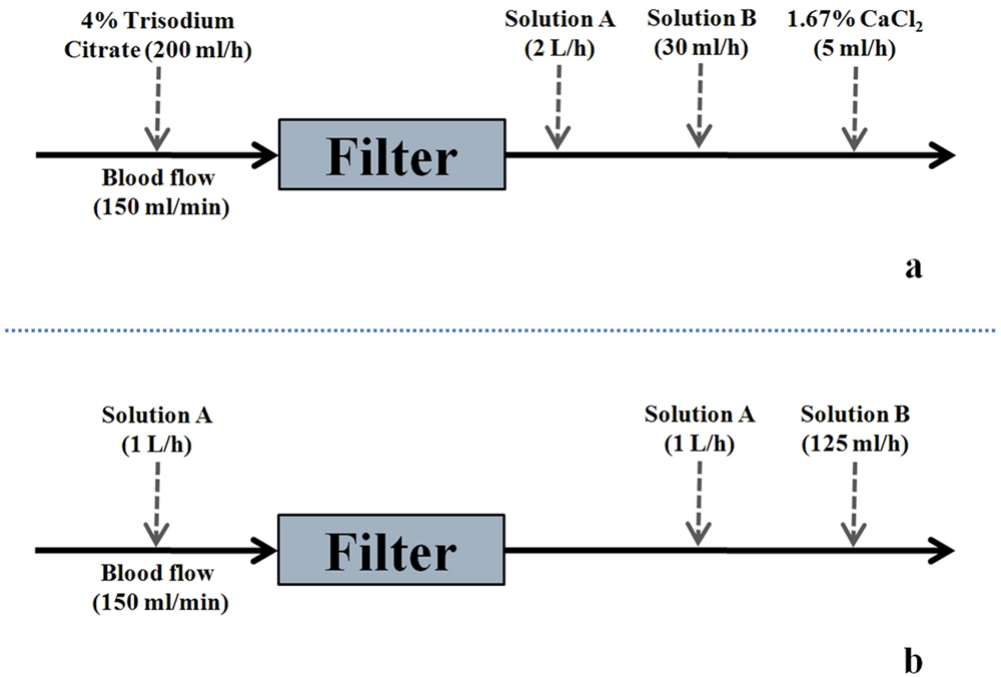
The two most employed modes in our center for CRRT in patients with a high risk of bleeding, i.e., RCA-CVVH (postdilution) using the Fresenius CiCa^®^ system (**a**) and CVVH with no anticoagulation (pre- and postdilution) (**b**). A commercial calcium-containing replacement fluid including Solution A and B (5% sodium bicarbonate) was used in CRRT. Some relative preset parameters are shown above.

Depending on the blood potassium level of patients and the clinical requirements, an appropriate amount of potassium chloride (KCl) was added to the replacement fluid according to the intensivist’s instruction. The potassium level would increase by 0.335 mmol/L when 1 ml of 10% KCl was infused into the replacement fluid (4 L). NaHCO_3_ (5%) was delivered via post-filter independently according to the systemic acid-base balance. A diagram was presented for two anticoagulation modes in the study during CRRT and their preset parameters (Fig. 7). The prescribed dose aimed for a target of an average effluent flowrate of 20–25 ml/kg/h. CaCl_2_ (1.67%, 113 mmol/L) was initially preset at 5 ml/h, corresponding to 0.3 mmol/L replacement fluid per hour. Citrate (4%, 136 mmol/L) was preset at 200 ml/h, corresponding to 3 mmol/L in blood with blood flow at 150 ml/min.

Under the Fresenius CiCa^®^ system during RCA-CVVH, the citrate infusion was finely adjusted according to the post-filter iCa level, and the CaCl_2_ (1.67%) infusion was adjusted based upon the systemic iCa level (Table 2). The amount of net fluid removal was left to the discretion of the intensivist but never exceeded 200 ml/h.

**Table 2:** Fine adjustment for RCA-CVVH.

Post-filter ionized calcium (mmol/L)	The change of citrate infusion (mmol/L)	Systemic ionized calcium (mmol/L)	The change of CaCl_2_ infusion (mmol/L)
>0.45	0.3 ↑	>1.45	0.6 ↓
0.41–0.45	0.2 ↑	1.31–1.45	0.4 ↓
0.36–0.40	0.1 ↑	1.21–1.30	0.2 ↓
0.25–0.35	No change	1.00–1.20	No change
0.20–0.24	0.1 ↓	0.90–0.99	0.2 ↑
0.15–0.19	0.2 ↓	<0.90	0.4 ↑
<0.15	0.3 ↓	—	—

Note: The citrate and CaCl_2_ infusion were increased (↑) or decreased (↓) by the indicated flowrate. The concentration (mmol/L) denotes the citrate level in the pre-filter blood and the CaCl_2_ level corresponding to the replacement fluid.

An appropriate amount of sodium chloride (NaCl, 10%) should be additionally infused into the replacement fluid for hypernatremia patients in both groups (Table 3). Blood sodium levels were controlled to be decreased by ≤10 mmol/L per day or ≤0.5 mmol/L per hour to prevent demyelination of nerve tissue.

**Table 3:** Protocol for hypernatremia patients in our centre.

<basic blood sodium (mmol/L)	additional 10% NaCl infusion (ml)^a^	theoretical sodium level of the fluid (mmol/L)^b^
145–149	9	144.6
150–154	21	149.4
155–159	32	153.8
160–164	45	158.9
165–170	56	163.2

^a^Extra sodium chloride (NaCl, 10%) was added to the replacement fluid (4.25 L) to avoid restoration of blood sodium intensively.

^b^Theoretical sodium concentration of the replacement fluid (4.25 L).

### Transfusion protocol

A restrictive strategy for blood transfusion was adopted in our center on the basis of the NICE guidelines (NG24). The infusion of packed red blood cells (PRBC) was initiated only when the hemoglobin level was lower than a threshold of 70 g/L, and the target for transfusion was 70–90 g/L. A higher hemoglobin threshold (80 g/L) and transfusion target (80–100 g/L) could be considered for patients with acute coronary syndrome. Prophylactic platelet transfusion should be considered in patients with a platelet count below 10 × 10^9^/L. A higher platelet threshold (30 × 10^9^/L) could be used for patients with clinically significant bleeding. The threshold for platelet transfusion was set minimally at 50 × 10^9^/L if patients with a high risk of bleeding underwent surgery or invasive procedures. Major hemorrhage events were defined as patients who were hemodynamically unstable because of blood loss, as evidenced by such conditions as a systolic blood pressure lower than 90 mmHg, a decreased hemoglobin concentration by more than 20 g/L within 24 h, or more than 400 ml of packed red blood cells required for blood transfusion.

### Clinical information record

Certain characteristics of the patients in both groups were collected, such as gender, age, weight, major diagnosis, comorbidities, acute physiology and chronic health evaluation II (APACHE II), sequential organ failure assessment (SOFA), length of ICU stay, length of hospital stay and causes of death. More information during CRRT was recorded, which included the effluent volume per hour, filter survival, causes of filter replacement, aggravated bleeding of organ or tissue and cardiac dysrhythmia. Blood tests for complete blood count, electrolytes, coagulation, liver and renal functions, blood gas analysis and core temperature were also monitored regularly. The levels of systemic tCa and iCa and post-filter iCa in the RCA group were analyzed at intervals of 4–8 h during CRRT. The expense of CRRT was also evaluated. The cost of the CiCa^®^ unit including the filter and the corresponding connection tubes was $421.90, while the cost of the general unit without CiCa^®^ was $265.60. The charge for 1 h of extracorporeal treatment was $12.50. The cost of the commercial solution of 4% trisodium citrate (200 ml) was $7.00. One U.S. dollar ($) was equivalent to 6.4 RMB.

### Citrate analysis

The citrate concentrations in the systemic plasma, post-filter plasma and ultrafiltrate were detected every 8 h during CRRT. The procedure for blood sample collection and pretreatment is briefly described below. Whole blood was immediately centrifuged at 3,000 rpm for 10 mins at 4 °C, and then the plasma was stored at −20 °C for the subsequent analysis in 48 h. Vials containing frozen plasma samples were thawed in 37 °C water, and 0.5 ml of plasma was acidified with 2 μl of 1 mol/L hydrochloric acid and then vortex-mixed for 1 min. Acidified plasma was transferred to the donor side of the Amicon^®^ Ultra-0.5 centrifugal filter devices (Millipore, USA) with a molecular weight cutoff of 10,000 D, followed by centrifugation at 14,500 rpm at 4 °C for 10 mins. One hundred microliters of the subnatant ultrafiltrate was injected into the HPLC system for analysis. Ultrafiltrate samples without preparation were analyzed with the same HPLC system. Separation was performed using an Atlantis^®^ T3 column (150 × 4.6 mm, 3 μm). The mobile phase was 30 mmol/L KH_2_PO_4_ with pH 2.8 at a flow rate of 0.8 ml/min. The wavelength for determination of the citrate was set at 210 nm. An area under the chromatographic peak was adopted for the quantification of the citrate level. The linearity of citrate determinations in the plasma and ultrafiltrate was good between 0.1 and 10 mmol/L, with a correlation coefficient of >0.998. The intraday and interday precisions were high, with a relative standard deviation (RSD) < 2.0% for all experimental results. The study involving the citrate level analysis was conducted in the Research Center of Analysis and Measurement, Zhejiang University of Technology, Hangzhou, China.

### Statistics

SPSS for Windows (Statistical Package for the Social Sciences, IBM SPSS Statistics 19, Chicago, IL, USA) was available for all statistical analyses. The independent samples *t*-test or one-way ANOVA was performed to compare continuous numeric variables with a normal distribution, which are presented as the means and 95% confidence interval. The Mann-Whitney *U* nonparametric test was used for comparing continuous numeric variables with a skewed distribution, which are shown as the medians and 95% confidence interval. The Pearson *χ*^2^ test or Fisher’s exact test (when necessary) was employed to compare the categorical variables, which are presented as frequencies and proportions (percentages). The Kaplan-Meier survival analysis using the log-rank test (Mantel-Cox test) was implemented for comparing the hemofilter lifespan within the two groups. A *p*-value of < 0.05 was considered to be statistically significant.
